# Statistical power of likelihood ratio and Wald tests in latent class models with covariates

**DOI:** 10.3758/s13428-016-0825-y

**Published:** 2016-12-30

**Authors:** Dereje W. Gudicha, Verena D. Schmittmann, Jeroen K. Vermunt

**Affiliations:** Department of Methodology and Statistics, Tilburg University, PO Box 90153, 5000LE Tilburg, The Netherlands

**Keywords:** Latent class, Power analysis, Likelihood ratio, Wald test, Asymptotic distributions, Non-centrality parameter, Large simulated data set

## Abstract

This paper discusses power and sample-size computation for likelihood ratio and Wald testing of the significance of covariate effects in latent class models. For both tests, asymptotic distributions can be used; that is, the test statistic can be assumed to follow a central Chi-square under the null hypothesis and a non-central Chi-square under the alternative hypothesis. Power or sample-size computation using these asymptotic distributions requires specification of the non-centrality parameter, which in practice is rarely known. We show how to calculate this non-centrality parameter using a large simulated data set from the model under the alternative hypothesis. A simulation study is conducted evaluating the adequacy of the proposed power analysis methods, determining the key study design factor affecting the power level, and comparing the performance of the likelihood ratio and Wald test. The proposed power analysis methods turn out to perform very well for a broad range of conditions. Moreover, apart from effect size and sample size, an important factor affecting the power is the class separation, implying that when class separation is low, rather large sample sizes are needed to achieve a reasonable power level.

## Introduction

In recent years, latent class (LC) analysis has become part of the standard statistical toolbox of researchers in the social, behavioral, and health sciences. A considerable amount of articles have been published in which LC models are used (a) to identify subgroups of subjects with similar behaviors, attitudes, or preferences, and (b) to investigate whether the respondents’ class memberships can be explained by variables such as age, gender, educational status, and type of treatment. This latter type of use is often referred to as LC analysis with covariates or concomitant variables. Example applications include the assessment of the effect of maternal education on latent classes differing in health behavior (Collins and Lanza 2010), of education and age on latent classes with different political orientations (Hagenaars and McCutcheon 2002), of age on latent classes of crime delinquencies (Van der Heijden et al. 1996), and of paternal occupation on latent classes with different gender-role attitudes (Yamaguchi 2000). Though most methodological aspects of the LC analysis with covariates are well addressed among others by Bandeen-Roche et al. (1997), Dayton and Macready (1988), Formann (1992), and Vermunt (1996), it is unclear how to perform power analysis when one plans to apply these models. This is a great omission since a study using an under-powered design may lead to an enormous waste of resources.

As in standard logistic regression analysis, hypotheses about the effects of covariates on the individuals’ latent class memberships can be tested using either likelihood ratio (LR), Wald, or score (Lagrange multiplier) tests (Agresti 2007). Under certain regularity conditions, these three test statistics are asymptotically equivalent, each following a central Chi-square distribution under the null hypothesis and a non-central Chi-square under the alternative hypothesis. In the manuscript, we focus on the Wald and LR tests. Researchers using such tests often ask questions such as: “What sample size do I need to detect a covariate effect of a certain size?” , “If I want to test the effect of a covariate, should I worry about the number and/or quality of the indicators used the LC model?” , and “Should I use a LR or a Wald test?” These questions can be answered by assessing the statistical power of the planned tests; that is, by investigating the probability of correctly rejecting a null hypothesis when the alternative is true. The aim of the current paper is to present power analysis methods for the LR and the Wald test in LC models with covariates, as well as to assess the data requirements for achieving an acceptable power level (say of .8 or larger). We also compare the power of the LR and the Wald test for a range of design and population characteristics.

Recently, power and sample size determination in LC and related models have received increased attention in the literature. Gudicha et al. (2016) studied the power of the Wald test for hypotheses on the association between the latent classes and the observed indicator variable(s), and showed that power is strongly dependent on class separation. Tein et al. (2013) and Dziak et al. (2014) studied the statistical power of tests used for determining the number of latent classes in latent profile and LC analysis, respectively. To the best of our knowledge, no previous study has yet investigated power analysis for LC analysis with covariates, nor compared the power of the LR and the Wald test in LC analysis in general.

Hypotheses concerning covariate effects on latent classes may be tested using either LR or Wald tests, but it is unknown which of these two types of tests is superior in this context. While the LR test is generally considered to be superior (see, for example, Agresti (2007) and Williamson et al. (2007)), the computational cost of the LR test will typically be larger because it requires fitting both the null hypothesis and the alternative hypothesis model, while the Wald test requires fitting only the alternative hypothesis model. Note that when using LR tests, a null hypothesis model should be estimated for each of the covariates, which can become rather time consuming given the iterative nature of the parameter estimation in LC models and the need to use multiple sets of starting values to prevent local maxima. A question of interest though is whether the superiority of the LR test is substantial enough to outweigh the computational advantages of the Wald test in the context of LC modeling with covariates.

For standard logistic regression analysis, various studies are available on power and sample-size determination for LR and Wald tests (Demidenko 2007; Faul et al. 2009; Hsieh et al. 1998; Schoenfeld and Borenstein 2005; Whittemore 1981; Williamson et al. 2007). Here, we not only build upon these studies but also investigate design aspects requiring special consideration when applying these tests in the context of LC analysis. A logistic regression predicting latent classes differs from a standard logistic regression in that the outcome variable, the individual’s class membership, is unobserved, but instead determined indirectly using the responses on a set of the indicator variables. This implies that factors affecting the uncertainty about the class memberships, such as the number of indicators, the quality of indicators, and the number of latent classes, will also affect the power and/or the required sample size.

In the next section, we introduce the LC model with covariates and discuss the LR and Wald statistics for testing hypotheses about the logit parameters of interest, present power computation methods for the LR and the Wald tests, and provide a numerical study illustrating the proposed power analysis methods. The paper ends with a discussion and conclusions.

## The LC model with covariates

Let *X* be the latent class variable, *C* the number of latent classes, and *c*=1,2,3,...,*C* the class labels. We denote the vector of *P* indicator variables by **Y**=(*Y*_1_,*Y*_2_,*Y*_3_,...,*Y*_*P*_), and the response of subject *i* (for *i*=1,2,3,...,*n*) to a particular indicator variable by *y*_*ij*_ and to all the *P* indicator variables by **y**_*i*_. Denoting the value of subject *i* for covariate *Z*_*k*_ (for *k*=1,2,3,...,*K*) by *z*_*ik*_, we define the LC model with covariate as follows:

1 $$ P(\mathbf{y}_{i}|\mathbf{z}_{i})={\sum}_{c=1}^{C}P(X=c|\mathbf{Z}=\mathbf{z}_{i}){\prod}_{j=1}^{P}P(Y_{j}=y_{ij}|X=c)  $$

where **z**_*i*_ is the vector containing the scores of subject *i* on the *K* covariates. The term *P*(*X* = *c*|**Z** = **z**_*i*_) represents the probability of belonging to class *x* given the covariate values **z**_*i*_, and *P*(*Y*_*j*_ = *y*_*ij*_|*X* = *c*) is the conditional probability of choosing response *y*_*ij*_ given membership of class *x*. The response variables **Y** in equation (1) could represent a set of symptoms related to certain types of psychological disorders, for example. In that case, the latent classes *X* would represent the disorder types. The covariates *Z*_*k*_ related to the prevalence of the latent classes or disorder types could be age and gender.

The LC model defined in equation (1) is based on the following assumptions. Firstly, we assume that the latent classes are mutually exclusive and exhaustive; that is, each individual is a member of one and only one of the *C* latent classes. The second assumption is the local independence assumption, which specifies that the responses to the indicator variables are independent given the class membership. For simplicity, we also assume that given the class membership, the covariates have no effect on the indicator variables.

The term *P*(*X* = *c*|**Z** = **z**_*i*_) in equation (1) is typically modeled by a multinomial logistic regression equation (Magidson and Vermunt 2004). Using the first class as the reference category, we obtain:

$$P(X=c|\mathbf{Z}=\mathbf{z}_{i})=\frac{\exp {(\gamma_{0c}+{\sum}_{k=1}^{K}\gamma_{kc}}z{_{ik})}}{1+{\sum}_{s=2}^{C}\exp {(\gamma_{0s}+{\sum}_{k=1}^{K}\gamma_{ks}}z{_{ik})}}, $$

where *γ*_0*c*_ represents an intercept parameter and *γ*_*kc*_ a covariate effect. For each covariate, we have *C*−1 effect parameters. Assuming that the responses *Y*_*j*_ are binary, the logistic model for *P*(*Y*_*j*_=1|*X* = *c*) may take on the following form:

$$P(Y_{j}=1|X=c)=\frac{\exp (\beta_{jc})}{1+\exp ({\beta_{jc}})}. $$

The *γ* parameters are sometimes referred to as the structural parameters, and the *β* parameters as the measurement parameters. We denote the full set of model parameters by Φ, which with binary responses is a column vector containing (*K*+1)(*C*−1) + *C*⋅*P* non-redundant parameters.

The parameters of the LC model with covariates are typically estimated by means of maximum likelihood (ML) estimation, in which the log-likelihood function

2 $$ l({\Phi} )={\sum}_{i=1}^{n}\log {P(\mathbf{y}_{i}|\mathbf{z}_{i})}  $$

is maximized using, for instance, the expectation maximization (EM) algorithm. Inference concerning the Φ parameters is based on the ML estimates $\hat {\Phi }$, which can be used for hypotheses testing or confidence interval estimation. In the current work, we focus on testing hypotheses about the ***γ*** parameters, the most common of which is testing the statistical significance for the effect of covariate *k* on the latent class memberships. The corresponding null hypothesis can be formulated as

$$H_{0}:\boldsymbol{\gamma}_{k}=0, $$

which specifies that the *γ*_*kc*_ values in $\boldsymbol {\gamma }_{k}^{^{\prime } }=(\gamma _{k2},\gamma _{k3},\gamma _{k4},...\gamma _{k(C)})$ are simultaneously zero.^1^ Using either the LR or the Wald test, the null significance of this hypothesis is tested against the alternative hypothesis:

$$H_{1}:\boldsymbol{\gamma}_{k}\neq 0. $$

Following Buse (1982) and Agresti (2007), we define the LR and the Wald statistic for this test as follows:

3 $$ \begin{array}{llll} & LR=2l(\hat{\Phi}_{1})-2l(\hat{\Phi}_{0}) \\ & W=\hat{\boldsymbol{\gamma} }_{k}^{^{\prime} }\mathbf{V}(\hat{\boldsymbol{ \gamma} }_{k})^{-1}\hat{\boldsymbol{\gamma} _{k}}, \end{array}  $$

where *l*(.) is the log-likelihood function as defined in Eq. 2, $\hat {\Phi }_{1}$ and $\hat {\Phi }_{0}$ are the ML estimates of Φ under the unconstrained alternative and constrained null model, respectively, $\hat {\boldsymbol {\gamma } }_{k}$ are the ML estimates for the logit coefficients of covariate *Z*_*k*_, and $\mathbf {V}(\hat {\boldsymbol {\gamma } }_{k})$ is the *C*−1 by *C*−1 covariance matrix of $\hat {\boldsymbol { \gamma } }_{k}$.

As we see from Eq. 3, the LR test for a covariate effect on the latent classes involves estimating two models: the *H*_0_ model with the covariate excluded and the *H*_1_ model with covariate included. The LR value is obtained as the difference in minus twice the log-likelihood values of these two models. The Wald test is a multivariate generalization of the *z*-test that makes the parameters comparable by dividing each element of a parameter by its standard deviation, which is equivalent to a one degree of freedom Chi-square test for *z* ^2^ (i.e., parameter squared divided by its variance). As can be seen, in the Wald formula we do the same but using the vector of parameters (which is squared) and the covariance matrix (by which we divide).

When multiple covariates are included in the logistic regression, quantities required to compute the power and sample size of the LR test is obtained by estimating the *H*_0_ model with all the covariates except the one we wanted to be tested included and the *H*_1_ model with all the covariates included. Whereas for the Wald test, we compute the expected information matrix from the *H*_1_ model with all the covariates included, and then correct the standard errors for correlation between covariates as suggested by Hsieh et al. (1998).

Large sample probability theory suggests that, under certain regularity conditions, if the null hypothesis holds, both the *LR* and *W* statistics asymptotically follow a central Chi-square distribution with *C*−1 degrees of freedom (see for example Agresti (2007), Buse (1982), and Wald (1943)). From this theoretical distribution, the *p* value can be obtained, and the null hypothesis should be rejected if this *p* value is smaller than the nominal type I error *α*.

## Power and sample-size computation

For power or sample-size computation, not only the distribution of the test statistic under the null hypothesis needs to be obtained but also its distribution under the alternative hypothesis. Under certain regularity conditions, if the alternative hypothesis holds, both the LR and the Wald statistic follow a non-central Chi-square distribution with *C*−1 degrees of freedom and non-centrality parameter *λ*:

4 $$ \begin{array}{lllll} & \lambda_{LR_{n}}=n\left( 2E[l({\Phi}_{1})]-2E[l({\Phi}_{0})]\right) \\ & \lambda_{W_{n}}=n\left( \boldsymbol{\gamma}_{k}^{^{\prime} }\mathbf{V}(\boldsymbol{\gamma} _{k})^{-1}\boldsymbol{\gamma}_{k}\right) . \end{array}  $$

Here, *E*[*l*(Φ_1_)] and *E*[*l*(Φ_0_)] denote the expected value of the log-likelihood for a single observation under the alternative and null model, respectively, assuming that the alternative model holds. In the definition of $\lambda _{W_{n}}$, **V**(***γ***_*k*_)^−1^ is the matrix of parameter covariances based on the expected information matrix for a single observation. Note that (4) is rather similar to equation (3). However, an important difference is that equation (3) represents the sample statistics (used for the actual testing) evaluated at the ML estimates computed using the sample concerned, whereas equation (4) gives the expected value of these statistics for a given sample size evaluated at the assumed population values for the parameters, and are thus not sample statistics.

The power of a test is defined as the probability that the null hypothesis is rejected when the alternative hypothesis is true. Using the theoretical distribution of the LR and Wald tests under the alternative hypothesis, we calculate this probability as

5 $$ \begin{array}{llllll} & power_{LR}=P\left( LR>\chi_{(1-\alpha )}^{2}(C-1)\right) \\ & power_{W}=P\left( W>\chi_{(1-\alpha )}^{2}(C-1)\right) , \end{array}  $$

where $\chi _{(1-\alpha )}^{2}(C-1)$ is the (1−*α*) quantile value of the central Chi-square distribution with *C*−1 degrees of freedom, and *LR* and *W* are random variates of the corresponding non-central Chi-square distribution. That is, *L* *R*,*W*∽*χ* ^2^(*C*−1,*λ*), where *λ* is as defined in Eq. (4). For the Wald test, this large sample asymptotic approximation requires multivariate normality of the ML estimates of the logit parameters, as well as that **V**(***γ***_*k*_) is consistently estimated by $\mathbf {V}\hat {\boldsymbol {\gamma }}_{k})$ (Redner 1981; Satorra and Saris 1985; Wald 1943).

Computing the asymptotic power (also called the theoretical power) using Eq. 5, requires us to specify the non-centrality parameter. However, in practice, this non-centrality parameter is rarely known. Below, we show how to obtain the non-centrality parameter using a large simulated data set, that is, a data set generated from the model under the alternative hypothesis.

### Calculating the non-centrality parameter

O’Brien (1986) and Self et al. (1992) showed how to obtain the non-centrality parameter for the LR statistic in log-linear analysis and generalized linear models using a so-called “exemplary ” data set representing the population under the alternative model. In LC analysis with covariates, such an exemplary data set would contain one record for each possible combination of indicator variable responses and covariate values, with a weight equal to the likelihood of occurrence of the pattern concerned. Creating such an exemplary data set becomes impractical with more than a few indicator variables, with indicator variables with larger numbers of categories, and/or when one or more continuous covariates are involved. As an alternative, we propose using a large simulated data set from the population under the alternative hypothesis. Though such a simulated data set will typically not include all possible response patterns, if it is large enough, it will serve as a good approximation of the population under *H*_1_.

By analyzing the large simulated data set using the *H*_0_ and *H*_1_ models, we obtain the values of the log-likelihood function under the null and alternative hypotheses. The large data set can also be used to get the covariance matrix of the parameters based on the expected information matrix. These quantities can be used to calculate the non-centrality parameters for the LR and Wald statistics as shown in equation (4). More specifically, the non-centrality parameter is calculated, using this large simulated data set, via the following simple steps:

1. Create a large data set by generating say *N*=1000000 observations from the model defined by the alternative hypothesis.
2. Using this large simulated data set, compute the maximum value of the log-likelihood for both the constrained null model and the unconstrained alternative model. These log-likelihood values are denoted by $\widetilde {l} ({\Phi }_{0})$ and $\widetilde {l}({\Phi }_{1})$ , respectively. For the Wald test, use the large simulated data to approximate the expected information matrix under the alternative model. This yields $\widetilde {\mathbf {V}}(\boldsymbol {\gamma } _{k})$, the approximate covariance matrix of ***γ***_*k*_.
3. The non-centrality parameter corresponding to a sample of size 1 is then computed as follows:
  $$\lambda_{LR_{1}}=\frac{2\widetilde{l}({\Phi}_{1})-2\widetilde{l}({\Phi}_{0})}{ N}\quad \text{and}\quad \lambda_{W_{1}}=\frac{\boldsymbol{\gamma}_{k}^{^{\prime} }\widetilde{\text{\textbf{V}}}(\boldsymbol{\gamma }_{k})^{-1} \boldsymbol{\gamma}_{k}}{N} $$
  for the LR and Wald test, respectively. As can be seen, this involves computing the LR and the Wald statistics using the information from step 2, and subsequently rescaling the resulting values to a sample size of 1.
4. Using the proportionality relation between sample size and non-centrality parameter as shown in Eq. 4, the non-centrality parameter associated with a sample of size *n* is then computed as $\lambda _{LR_{n}}=n\lambda _{LR_{1}}\text { and} \lambda _{W_{n}}=n\lambda _{W_{1}}$ (Brown et al. 1999; McDonald and Marsh 1990; Satorra and Saris 1985).

### Power computation

The power computation itself proceeds as follows:

1. Given the assumed population values under the alternative hypothesis, compute the non-centrality parameter *λ*_1_ using the large simulated data set as discussed above. Rescale the non-centrality parameter to the sample size under consideration.
2. For a given type I error *α*, read the (1−*α*) quantile value from the (central) Chi-square distribution with *C*−1 degrees of freedom. That is, find $\chi _{(1-\alpha )}^{2}(C-1)$ such that $ P\left (LR>\chi _{(1-\alpha )}^{2}(C-1)\right ) =\alpha $ and $P\left (W>\chi _{(1-\alpha )}^{2}(C-1)\right ) =\alpha $ for the LR and Wald test statistics, respectively. This quantile—also called the critical value—can be read from the (central) Chi-square distribution table, which is available in most statistics text books. For example, for *α*=.05 and *C*=2, we have $\chi _{(.95)}^{2}(1)=3.84$ (Agresti 2007).
3. Using the non-centrality parameter value obtained in step 1, the specified sample size *n*, and the critical value obtained in step 2, evaluate Eq. 5 to obtain the power of the LR or Wald test of interest. This involves reading the probability concerned from a non-central Chi-square distribution with degrees of freedom *C*−1 and non-centrality parameter *λ*_*n*_.

### Sample-size computation

The expression for sample-size computation can be derived from the relation in Eq. 4:

6 $$ \begin{array}{llllll} & n_{LR}=\lambda_{n}\left\{ 2E[l({\Phi}_{1})]-2E[l({\Phi}_{0})]\right\}^{-1} \\ & n_{W}=\lambda_{n}\left[ \boldsymbol{\gamma}_{k}^{^{\prime} }\mathbf{V}(\boldsymbol{\gamma} _{k})^{-1}\boldsymbol{\gamma}_{k}\right]^{-1}, \end{array}  $$

where *n*_*LR*_ and *n*_*W*_ are the LR and Wald sample size, respectively.

Using equation (6), the sample size required to achieve a specified level of power is computed as follows:

1. For a given value of *α*, read the (1−*α*) quantile value from the central Chi-square distribution table.
2. For a given power and the critical value obtained in step 1, find the non-centrality parameter *λ*_*n*_ such that, under the alternative hypothesis, the condition that the power is equal to $P\left (LR>\chi _{(1-\alpha )}^{2}(C-1)\right ) $ for the LR statistic and $P\left (W>\chi _{(1-\alpha )}^{2}(C-1)\right ) $ for the Wald statistic is satisfied.
3. Given the parameter values of the model under the alternative hypothesis and the *λ*_*n*_ value obtained in step 2, use Eq. (6) to compute the required sample size. Note that also for sample size computation a large simulated data set is used to approximate *E*[*l*(Φ_0_)], *E*[*l*(Φ_1_)], and **V** (***γ***).

### LC-specific factors affecting the power

As in any statistical model, also in LC analysis the power of a test is influenced by sample size, effect size, and type I error. However, an important difference between a LC analysis with covariates and a standard logistic regression analysis is that in the former the outcome variable in the logistic regression model is not directly observable, and thus its value is uncertain. It can, therefore, be expected that also factors affecting the certainty about individuals’ class memberships (or the class separation) will affect the power of the statistical tests of interest. Information on the (un)certainty about individuals’ class memberships is contained in the posterior membership probabilities:

7 $$ P(X\,=\,c|\mathbf{y}_{i},\mathbf{z}_{i})\,=\,\frac{P(X=c|\mathbf{z} _{i}){\prod}_{j=1}^{P}P(Y_{j}=y_{ij}|X=c)}{{\sum}_{s=1}^{C}P(X=s|\mathbf{z}_{i}){\prod}_{j=1}^{P}P(Y_{j}=y_{ij}|X=s)}.  $$

Gudicha et al. (2016) discussed how the elements of the expected information matrix for class-indicator associations are related to the posterior class membership probabilities; that is, the diagonal elements become smaller when the posterior membership probabilities are further away from 0 and 1. A similar thing applies to the covariate effects. When covariates are included, the diagonal element of the information matrix for effect *γ*_*kc*_ conditional on **y** and **z** can be expressed as follows:

8 $$\begin{array}{@{}rcl@{}} {I}_{(\gamma_{kc},\gamma_{kc}|\mathbf{y,z})}\!\!&=&\!\!\left( z_{k}\right)^{2}\!\left\{ \left[ P(X\,=\,c|\mathbf{y},\mathbf{z})\right]^{2}\,-\,2P(X\,=\,c|\mathbf{y} ,\mathbf{z})P(X\,=\,c|\mathbf{z})\right.\\ &&\qquad\left.+\left[ P(X=c|\mathbf{z})\right]^{2}\right\} \end{array} $$

This reduces to the expression for the standard multinomial regression model when class membership is certain, that is, when *P*(*X* = *c*|**y**,**z**) equals 1 for one class and 0 for the others. It is mainly the term [*P*(*X* = *c*|**y**,**z**)]^2^ in Equation (8) which yields the information loss. The sum of this term over classes, and thus also the total information contributed by a data pattern, decreases when uncertainty about the class membership increases. This affects not only the power of the Wald test through the parameter covariance matrix but also the power of the LR test. A large amount of information on the parameters corresponds to a larger curvature of the log-likelihood function at $\hat {\Phi }_{1}$ (Buse 1982), which implies the difference between $2l(\hat { {\Phi }}_{1})$ and $2l(\hat {\Phi }_{0})$ will be larger. This will have a direct effect on both the LR value calculated via Eq. (3) and the non-centrality parameter calculated via the procedures discussed above.

Considering different scenarios for the LC model structure and parameter values, Gudicha et al. (2016) showed that more favorable conditions in terms of class separation occur with response probabilities which differ more across the classes, with a larger number of indicators, with more equal classes sizes, and with a smaller number of classes.

## Numerical study

The purpose of this numerical study is to (1) compare the power of the Wald test with the power of the LR test, (2) investigate the effect of factors influencing the uncertainty about the individuals’ class membership—mainly the measurement parameters—on the power of the Wald and LR tests concerning the structural parameters, (3) evaluate the quality of the power estimation using the non-centrality parameter value obtained with the large simulated data set, and (4) give an overview of the sample sizes required to achieve a power level of .8 or higher, .9 or higher, or .95 or higher in several typical study designs. In the current numerical study, we consider models with one covariate only, but the proposed methods are also applicable with multiple covariates. We assume asymptotic distributions for both the tests, and estimate the non-centrality parameter of the non-central Chi-square distribution using the large data set method described earlier. All analyses were done using the syntax module of the Latent GOLD 5.0 program (Vermunt and Magidson 2013).

### Study setup

The power of a test concerning the structural parameters is expected to depend on three key factors: the population structure and the parameter values for the other parts of the model, the effect sizes for the structural parameters to be tested, and the sample size. Important elements of the first factor include the number of classes, the number of indicator variables, the class-specific conditional response probabilities, and the class proportions (Gudicha et al. 2016). In this numerical study, we varied the number of classes (*C*=2 or 3) and the number of indicator variables (*P*=6 or 10). Moreover, the class-specific conditional response probabilities were set to 0.7, 0.8, or 0.9 (or, depending on the class, to 1–0.7, 1–0.8, and 1–0.9), corresponding to conditions with weak, medium, and strong class-indicator associations. The conditional response probabilities were assumed to be high for class 1, say 0.8, and low for class *C*, say 1–0.8, for all indicators. In class 2 of the three-class model, the conditional response probabilities are high for the first half and low for the second half of the indicators.

The effect size was varied for the structural parameters to be tested, that is, for the logit coefficients that specify the effect of a continuous covariate *Z* on the latent class memberships (see Eq. 2 above). Using the first class as the reference category, the logit coefficients were set to 0.15, 0.25, and 0.5, representing the three conditions of small, medium, and large effect sizes. In terms of the odds ratio, these small, medium, and large effect sizes take on the values 1.16, 2.28, and 1.65, respectively. Two conditions were used for the intercept terms: in the zero intercept condition, the intercepts were set to zero for both *C*=2 and *C*=3, while in the non-zero intercept condition the intercepts equaled -1.10 for *C*=2, and -1.10 and -2.20 for *C*=3. Note that the zero intercept condition yields equal class proportions (i.e., .5 each for *C*=2 and .33 each for *C*=3), whereas the non-zero intercept condition yields unequal class proportions (i.e., .75 and .25 for *C*=2, and .69, .23, and .08 for *C*=3).

In addition to the above-mentioned population characteristics, we varied the sample size (*n*=200, 500, or 1000) for the power computations. Likewise, for the sample-size computations, we varied the power values (*p* *o* *w* *e* *r*=.8, .9, or .95). The type I error was fixed to .05 in all conditions.

Gudicha et al. (2016) showed that a study design with low separation between classes leads to low statistical power of tests concerning the measurement parameters in a LC model. Therefore, Table 1 shows the entropy R-square,^2^ which measures the separation between classes for the design conditions of interest.

**Table 1:** The computed entropy R-square for different design cells

		Equal class proportions			Unequal class proportions		
		Class-indicator			Class-indicator		
		associations			associations		
		Weak	Medium	Strong	Weak	Medium	Strong
*C*=2	*P*=6	.574	.855	.981	.534	.838	.978
*C*=2	*P*=10	.732	.935	.997	.704	.944	.998
*C*=3	*P*=6	.354	.650	.900	.314	.618	.878
*C*=3	*p*=10	.502	.805	.969	.462	.782	.963

*C*= the number of classes; *P*= number of indicator variables. The entropy R-square values reported in this table pertain to the model with small effect sizes for the covariate effects, and these entropy R-square values slightly increase for the case when we have larger effect sizes

### Results

Tables 2, 3, and 4 present the power of the Wald and LR tests for different sample sizes, class-indicator associations, number of indicator variables, class proportions, and effect sizes. Several important points can be noted from these tables. Firstly, the power of the Wald and LR tests increases with sample size and effect size, which is also the case for standard statistical models (e.g., logistic regression for an observed outcome variable). Secondly, specific to LC models, the power of these tests is larger with stronger class-indicator associations, a larger number of indicator variables, and more balanced class proportions. These LC-specific factors affect the class separations as well, as can be seen from Table 1. Comparing the power values in Tables 2 and 3, we also observe that the statistical power of the tests depends on the number of classes as well. Thirdly, the power of the LR test is consistently larger than of the Wald test, though in most cases differences are rather small.

**Table 2:** The power of the Wald and the likelihood ratio test to reject the null hypothesis that covariate has no effect on class membership in the two-class latent class model; the case of equal class proportions

		*n*=200			*n*=500			*n*=1000		
Effect		Class-indicator			Class-indicator			Class-indicator		
size		associations			associations			associations		
		Weak	Medium	Strong	Weak	Medium	Strong	Weak	Medium	Strong
Six indicator variables										
Small	Wald	.125	.164	.181	.242	.338	.379	.429	.587	.645
	LR	.126	.166	.180	.245	.343	.377	.434	.594	.645
Medium	Wald	.269	.363	.408	.546	.721	.779	.835	.945	.971
	LR	.260	.369	.411	.548	.729	.784	.836	.953	.973
Large	Wald	.702	.868	.913	.976	.998	1	1	1	1
	LR	.743	.885	.923	.985	.998	1	1	1	1
Ten indicator variables										
Small	Wald	.147	.177	.184	.297	.369	.385	.523	.633	.655
	LR	.151	.176	.181	.307	.367	.380	.539	.63	.647
Medium	Wald	.319	.397	.412	.653	.766	.786	.914	.967	.974
	LR	.315	.402	.422	.647	.773	.796	.91	.969	.976
Large	Wald	.812	.903	.917	.994	.999	.999	1	1	1
	LR	.837	.918	.9309	.996	.999	.999	1	1	1

The power values reported in this table are obtained by assuming theoretical Chi-square distributions for both the Wald and the likelihood ratio test statistics, for which the non-centrality parameter of the non-central Chi-square is approximated using a large simulated data set

**Table 3:** The power of the Wald and the likelihood ratio test to reject the null hypothesis that the covariate has no effect on class membership in the three-class latent class model; the case of equal class proportions

		*n*=200			*n*=500			*n*=1000		
Effect size		Class-indicator associations			Class-indicator associations			Class-indicator associations		
		Weak	Medium	Strong	Weak	Medium	Strong	Weak	Medium	Strong
Six indicator variables										
Small	Wald	.081	.106	.125	.131	.200	.252	.222	.365	.464
	LR	.080	.108	.126	.130	.206	.255	.221	.377	.471
Medium	Wald	.135	.214	.272	.281	.478	.599	.517	.789	.894
	LR	.140	.215	.272	.295	.48	.600	.540	.792	.894
Large	Wald	.365	.642	.779	.752	.967	.994	.968	1	1
	LR	.436	.686	.810	.837	.978	.996	.989	1	1
Ten indicator variables										
Small	Wald	.089	.118	.130	.155	.233	.265	.272	.430	.49
	LR	.092	.119	.133	.163	.236	.274	.289	.436	.504
Medium	Wald	.163	.252	.287	.353	.559	.628	.632	.864	.913
	LR	.178	.263	.290	.391	.583	.632	.686	.882	.915
Large	Wald	.471	.738	.807	.871	.989	.996	.994	1	1
	LR	.571	.772	.823	.938	.993	.997	.999	1	1

The power values reported in this table are obtained by assuming theoretical Chi-square distributions for both the Wald and the likelihood ratio test statistics, for which the non-centrality parameter of the non-central Chi-square is approximated using a large simulated data set

**Table 4:** The power of the Wald and the likelihood ratio test to reject the null hypothesis that the covariate has no effect on class membership; the case of unequal class proportions, and six indicator variables

		*n*=200			*n*=500			*n*=1000		
Effect size		Class-indicator associations			Class-indicator associations			Class-indicator associations		
		Weak	Medium	Strong	Weak	Medium	Strong	Weak	Medium	Strong
Two-class model										
Small	Wald	.102	.133	.148	.183	.263	.299	.319	.465	.525
	LR	.103	.136	.153	.185	.268	.312	.322	.475	.547
Medium	Wald	.195	.283	.322	.411	.590	.658	.688	.872	.918
	LR	.197	.282	.331	.414	.590	.674	.693	.871	.926
Large	Wald	.549	.761	.826	.909	.988	.996	.995	1	1
	LR	.590	.783	.844	.933	.991	.997	.998	1	1
Three-class model										
Small	Wald	.077	.100	.120	.120	.185	.238	.198	.334	.439
	LR	.076	.101	.121	.119	.188	.242	.197	0.34	.447
Medium	Wald	.125	.197	.257	.253	.439	.570	.467	.746	.873
	LR	.127	.208	.267	.257	.465	.593	.474	.775	.889
Large	Wald	.337	.600	.751	.712	.951	.990	.945	.999	1
	LR	.387	.641	.785	.782	.966	.994	.977	1	1

The power values reported in this table are obtained by assuming theoretical chi-square distributions for both the Wald and the likelihood ratio test statistics, for which the non-centrality parameter of the non-central Chi-square is approximated using a large simulated data set

The results in Tables 2, 3, and 4 further suggest that, for a given effect size, a desired power level of say .8 or higher can be achieved by using a larger sample, more indicator variables, or, if possible, indicator variables that have a stronger association with the respective latent classes. Given a set of often-unchangeable population characteristics (e.g., the class proportions, the class conditional response probabilities, and the effect sizes of the covariate effects on latent class memberships), one will typically increase the power by increasing the sample size. Table 5 presents the required sample size for the Wald test to achieve a power of .8, .9, and .95 under the investigated conditions. As can be seen from Table 5, for the situation where the class proportions are equal, the number of response variables is equal to 6, the number of classes is equal to 2, and the class-indicator associations are strong, a power of 0.80 or higher is achieved (1) for a small effect size, using a sample of size 1434, (2) for a medium effect size, using a sample of size 527, and (3) for a large effect size, using a sample of size 143. When the class-indicator associations are weak, the class proportions are unequal, or the requested power is .9, the required samples become larger. We also observe from the same table that in three-class LC models with six indicator variables and strong class-indicator associations, a power of .80 or higher is achieved by using sample sizes of 2120, 777, and 210, for small, medium, and large effect sizes, respectively.

**Table 5:** Sample-size requirements for Wald statistic in testing the covariate effect on class membership given specified power levels, class-indicator associations, number of indicator variables, number of classes, class proportions, and effect sizes

	*p* *o* *w* *e* *r*=.8			*p* *o* *w* *e* *r*=.9			*p* *o* *w* *e* *r*=.95		
Effect size	Class-indicator			Class-indicator			Class-indicator		
	associations			associations			associations		
	Weak	Medium	Strong	Weak	Medium	Strong	Weak	Medium	Strong
Two-class model with equal class proportions and six indicator variables									
Small	2473	1652	1434	3312	2210	1925	4097	2734	2380
Medium	911	606	527	1210	811	705	1509	1003	872
Large	253	165	143	338	221	191	418	273	236
Two-class model with equal class proportions and ten indicator variables									
Small	1929	1485	1412	2582	1988	1891	3193	2458	2338
Medium	709	544	518	949	729	693	1173	901	857
Large	194	148	140	260	198	188	321	245	232
Two-class model with unequal class proportions and six indicator variables									
Small	3544	2241	1916	4745	3000	2566	5868	3710	3173
Medium	1306	811	700	1749	1098	937	2163	1357	1159
Large	362	221	187	484	295	250	599	365	310
Three-class model with equal class proportions and six indicator variables									
Small	4922	2785	2120	6464	3657	2786	7888	4463	3400
Medium	1869	1025	777	2454	1347	1020	2995	1644	1245
Large	558	283	210	733	372	276	895	454	337

To assess the accuracy of the proposed power-analysis method, we also calculated the empirical power by Monte Carlo simulation. Using the critical value from the theoretical central Chi-square distribution, we computed the empirical power as the proportion of the *p* values rejected in 5000 samples generated from the population under the alternative hypothesis. In Table 6, we refer to this empirical power as ’LR empirical’ and ’Wald empirical’, indicating the power values computed from the empirical distribution of the LR and Wald statistics under the alternative hypothesis. We report results for the study conditions with a small effect size and equal class proportions, but similar results were obtained for the other conditions. Comparison of the theoretical with the corresponding empirical power values shows that these are very close in most cases, meaning that the approximation of the non-centrality parameter using the large simulated data set works well. Overall, the differences between the theoretical and empirical power values are small, with a few exceptions, which are situations in which the power is very low anyway. The exceptions occur when the class-indicator associations are weak in two-class LC models with six indicator variables and in three-class LC models with six as well as ten indicator variables, which in Table 1 correspond to the design conditions with entropy R-square values of .574, .345, and .502, respectively.

**Table 6:** Theoretical versus empirical (*H*_1_-simulated) power values of the likelihood ratio test of the covariate effect on class membership in design conditions of interest

	*n*=200			*n*=1000		
	Class-indicator			Class-indicator		
	associations			associations		
	Weak	Medium	Strong	Weak	Medium	Strong
Two-class model with six indicator variables						
Wald theoretical	.125	.164	.181	.429	.587	.645
Wald empirical	.131	.156	.176	.429	.584	.648
LR theoretical	.126	.166	.180	.434	.594	.645
LR empirical	.138	.177	.182	.432	.58	.648
Two-class model with ten indicator variables						
Wald theoretical	.147	.177	.184	.523	.633	.655
Wald empirical	.138	.175	.196	.513	.632	.652
LR theoretical	.151	.176	.181	.539	.63	.647
LR empirical	.150	.179	.189	.537	.638	.665
Three-class model with six indicator variables						
Wald theoretical	.081	.106	.125	.222	.365	.464
Wald empirical	.187	.134	.123	.223	.368	.454
LR theoretical	.08	.108	.126	.221	.377	.471
LR empirical	.238	.146	.134	.267	.374	.456
Three-class model with ten indicator variables						
Wald theoretical	.089	.118	.130	.272	.430	.490
Wald empirical	.169	.118	.127	.283	.426	.508
LR theoretical	.092	.119	.133	.289	.436	.504
LR empirical	.161	.133	.134	.286	.443	.493

The power values reported in this table are for the study design conditions with small effect size and equal class proportions

## Conclusions and discussion

Hypotheses concerning the covariate effects on latent class membership are tested using a LR, Wald, or score (Lagrange multiplier) test. In the current study, we presented and evaluated a power-analysis procedure for the LR and the Wald tests in latent class analysis with covariates. We discussed how the non-centrality parameter involved in the asymptotic distributions of the test statistics can be approximated using a large simulated data set, and how the value of the obtained non-centrality parameter can subsequently be used in the computation of the asymptotic power or the sample size.

A numerical study was conducted to study how data and population characteristics affect the power of the LR test and the Wald test, to compare the power of these two tests, and to evaluate the adequacy of the proposed power-analysis method. The results of this numerical study showed that, as in any other statistical model, the power of both tests depends on sample size and effect size. In addition to these standard factors, the power of the investigated tests depends on factors specific to latent class models, such as the number of indicator variables, the number of classes, the class proportions, and the strength of the class-indicator associations. These latent class-specific factors affect the separation between the classes, which we assessed using the entropy R-square value.

We saw that the sample size required to achieve a certain level of power depends strongly on the latent class-specific factors. The stronger the class-indicator variable associations, the more indicator variables, the more balanced the class proportions, and the smaller the number of latent classes, the smaller the required sample size that is needed to detect a certain effect size with a power of say .8 or higher. We can describe the same finding in terms of the entropy R-square, that is, the larger the entropy R-square, the smaller the sample size needed to detect a certain effect size with a power of say .8 or higher. A more detailed finding is that for a given effect size, the improvement in power obtained through adding indicator variables is more pronounced when class-indicator associations are weak or medium than when they are strong.

In line with previous studies (see for example Williamson et al. (2007)), the power for the LR test is larger than for the Wald test, though the difference is rather small. An advantage of the Wald test is, however, that it is computationally cheaper. Given the population values under the alternative hypothesis and the corresponding non-centrality parameter, the sample size for the Wald test can be computed using equation (6) directly. When using the LR test, the log-likelihood values under both the null hypothesis and the alternative hypothesis must be computed, which can be somewhat cumbersome when a model contains multiple covariates.

The adequacy of the proposed power analysis method was evaluated by comparing the asymptotic power values with the empirical ones. The results indicated that the performance of the proposed method is generally good. In the study design condition for which the entropy R-square is low—this occurs when few indicator variables with weak associations with the latent classes are used—and the sample size is small, the empirical power seemed to be larger than the asymptotic power, but these were situations in which the power turned out to be very low anyway. We also looked at the type I error rates of the Wald and LR tests (Table 7). In simulation conditions with medium/strong class-indicator associations or larger sample sizes, the type I error rates of the two tests are generally comparable and moreover close to the nominal level. However, in conditions with weak class-indicator association and small sample size, the type I error rates of both the tests are highly inflated. In such design conditions, instead of relying on the asymptotic results, we suggest using the empirical distributions constructed under the null and under the alternative hypothesis.

**Table 7:** Type I error rates for the Wald and LR tests

Sample	Test	Class-indicator associations		
Size	Statistic	Weak	Medium	Strong
200	Wald	.106	.077	.063
	LR	.204	.079	.062
500	Wald	.094	.072	.063
	LR	.118	.064	.056
1000	Wald	.08	.069	.061
	LR	.088	.068	.052

The type I error rates reported in this table pertain to the three-class model with six indicator variables and equal class size

We presented the large data set power analysis method for a simple LC model with cross-sectional data, but the same method may be applied with LC models for longitudinal and multilevel data. Moreover, although the simulations in the current paper were performed with a single covariate, it is expected that increasing the number of (uncorrelated) covariates to two or more will improve the entropy R-square and therefore also the power. The method may also be generalized to the so-called three-step approach for the analysis of covariate effects on LC memberships (Bakk et al. 2013; Gudicha and Vermunt 2013; Vermunt 2010).

As in standard logistic regression analysis (Agresti 2007), null hypothesis significance testing can be performed using Wald, likelihood ratio, or score (Lagrange multiplier) tests. Under certain regularity conditions, these three test statistics are asymptotically equivalent, each following a central Chi-square distribution under the null hypothesis and a non-central Chi-square under the alternative hypothesis. In the manuscript, we focus on the Wald and LR tests. Future research may consider extending the proposed power analysis method to the score test.

Sometimes researchers would like to know what the required effect size is for a specified sample size and power level (Dziak et al. 2014). Because our power and sample size computation methods depend on the alternative hypothesis, they cannot be used directly for such an effect-size computation. An indirect approach, however, can be used, which involves applying the method multiple times with different effect sizes. That is, if for the specified effect size and power level the computed sample size turns out to be larger than the sample size one would wishes to use, the effect size should be increased. If the computed sample size is smaller than one would like to use, the effect size can be reduced. Interpolation techniques can be used for an efficient implementation of such a search procedure.

This research has several practical implications. Firstly, it provides an overview of the design requirements for achieving a certain level of power in LC analysis with a covariate affecting class memberships. Secondly, it presents a tool for determining the required sample size given the specific research design that a researcher has in mind instead of relying on a rule of thumb. Based on the literature and on the results of our study, we can conclude that easy rules of thumb, such as a sample size of 500 suffices when the number of indicator variables is six, cannot be formulated for LC analysis.

## Appendix: : Latent GOLD syntax for Wald and LR power computations

This appendix illustrates the application of the proposed Wald and LR power computation methods using the Latent GOLD 5.0 program (Vermunt and Magidson 2013) Syntax . As an example, we use a two-class LC model with six binary response variables (*y*_1_ through *y*_6_) and a covariate (*Z*). Using the proposed methods, in order to perform a power computation, one should first create a small “example” data set; that is, a data set with the structure of the data one is interested in. With six binary response variables and one covariate, this file could be of the form:

This data file contains ten arbitrary values for the response variables, (standardized) values for the covariate, and the cases weights.

A Latent GOLD syntax model consists of three sections: “options ” “variables ” and “equations ”Ṫhe relevant LC model is defined as follows:

The “output ” option indicates that we wish to use dummy coding for the logit parameters with the first category as the reference category. Subsequently, we define the variables that are part of the model.

The two equations represent the logit equations for the structural and the measurement part of the model, respectively. Note that “1 ” indicate an intercept, and “ | ” that the intercept depends on the variable concerned. Next the power computation is proceeded as follow: Step 1: Using the large data set method, one should first simulate a large data set from the population defined by the *H*_1_ model. Simulating the large data set is done as follows:

In the “variables ” section, we define the variables which are in the model and also their number of categories. These are the six response variables, the latent variable “class ” , and the covariate *Z* . The “equations” section specifies the logit equations defining the model of interest, as well as the values of the population parameters. We use the “outfile ” option to indicate that a data file should be simulated, use the “caseweight ” to indicate the size of the large data set (here 1000000), and specify the parameter values of the population model. Note that the values .000, .25, and 0.84729786 for a logit coefficients corresponds to equal class size, medium effect size, and a conditional response probability of .70.

Step 2: Analyze the large data set obtained under step 1 using both the *H*_0_ and *H*_1_ model.

i) Fit the *H*_1_ model
ii) Fit the *H*_0_ model

Next, based on the results in (i) and (ii) for the LR test and the results in (i) for the Wald test, we compute the non-centrality parameter. Once the non-centrality parameter is obtained, one may use the following R subscript to compute the power:

where, in this example, the non-centrality parameter is equal to 1.7218

For the Wald test, power may also computed the power (without simulating the large data set) as follows.

The “output” line in the “options” section lists the output requested. With WaldPower= <*number*>, one requests a power or sample size computation. When using a “number” between 0 and 1, the program reports the required sample size for that power, and when using a value larger than 1, the program reports the power obtained with that sample size. The optional statement WaldTest=‘filename’ can be used to define the null hypothesis.

## Footnotes

1 For parameter identification, the logit parameter associated with the reference category is set to zero, resulting in *C*−1 non-redundant *γ* parameters. Note also that $\boldsymbol {\gamma }^{^{\prime } }$ denotes the transpose of a column vector ***γ***.

2 The entropy R-square compares the entropy of the specified model with the entropy of a baseline model in which the class proportions are the same for each individual. Latent Gold uses a baseline model based on the actual class proportions, whereas the others (e.g., Mplus and PROC LCA ) use a baseline model with equal class proportions. The Latent Gold R-square is a bit more conservative, but matches somewhat better the definition of an R-square: the improvement in prediction compared to an intercept only model.
